# Fruit Extract, Rich in Polyphenols and Flavonoids, Modifies the Expression of *DNMT* and *HDAC* Genes Involved in Epigenetic Processes

**DOI:** 10.3390/nu15081867

**Published:** 2023-04-13

**Authors:** Ghodratollah Nowrasteh, Afshin Zand, László Bence Raposa, László Szabó, András Tomesz, Richárd Molnár, István Kiss, Zsuzsa Orsós, Gellért Gerencsér, Zoltán Gyöngyi, Tímea Varjas

**Affiliations:** 1 Department of Public Health Medicine, Medical School, University of Pécs, 7624 Pécs, Hungary; ghodratollah.nowrasteh@medcarehospital.com (G.N.); af.zand@gmail.com (A.Z.); laszlo.szabo.pte@gmail.com (L.S.); tomeszandras@gmail.com (A.T.); richard.molnar.pte@gmail.com (R.M.); istvan.kiss@aok.pte.hu (I.K.); zsuzsa.orsos@aok.pte.hu (Z.O.); gellert.gerencser@gmail.com (G.G.); vtimi_68@yahoo.com (T.V.); 2 Faculty of Health Sciences, University of Pécs, 7621 Pécs, Hungary; bence.raposa@etk.pte.hu

**Keywords:** *DNMT*s, *HDAC*s, flavonoids, fruit extract, CD1 mice, qRT-PCR, gene expression

## Abstract

Recently, the field of epigenetics has been intensively studied in relation to nutrition. In our study, the gene expression patterns of histone deacetylases (*HDAC*s), which regulate the stability of histone proteins, and DNA methyltransferases (*DNMT*s), which regulate DNA methylation, were determined in mice. The animals were fed a human-equivalent dose of the aqueous extract of fruit seeds and peels, which is rich in flavonoids and polyphenols, for 28 days and then exposed to the carcinogen 7,12-dimethylbenz(a)anthracene (DMBA). The concentrations of trans-resveratrol and trans-piceid were determined in the consumed extract by HPLC and were 1.74 mg/L (SD 0.13 mg/L) and 2.37 mg/L (SD 0.32 mg/L), respectively, which corresponds to the consumption of 0.2–1 L of red wine, the main dietary source of resveratrol, in humans daily. Subsequently, 24 h after DMBA exposure, the expression patterns of the *HDAC* and *DNMT* genes in the liver and kidneys were determined by qRT-PCR. The DMBA-induced expression of the tested genes *HDAC1*, *HDAC2*, *DNMT1*, *DNMT3A* and *DNMT3B* was reduced in most cases by the extract. It has already been shown that inhibition of the *DNMT* and *HDAC* genes may delay cancer development and tumour progression. We hypothesise that the extract studied may exert chemopreventive effects.

## 1. Introduction

Malignant diseases are associated with high mortality rate, and identifying novel methods of reducing the development and progression of these diseases is of high interest. Recently, epigenetic changes caused by environmental factors, including nutrition, have been intensively studied [1]. Substances in food can alter the normal methylation patterns of DNA, resulting in either the abnormal inactivation or activation of genes, both of which can lead to cancer progression.

The methylation status of DNA, especially within promoter regions, can have notable effects on both the incidence and progression of many types of malignant diseases. Aberrant methylation patterns have been observed in almost all neoplasms, suggesting that DNA methylation may serve as an important molecular marker for cancer prevention, prognosis, and therapies [1,2,3]. Histone acetyltransferases (*HAT*s) and histone deacetylases (*HDAC*s) play essential roles in the epigenetic regulation of gene expression by determining the acetylation status of histone proteins, which determines whether chromatin has a “relaxed” or ”condensed” structure. Acetylation results in condensed structure and less active genes [4]. HDAC inhibitors (HDACis) exhibit antitumor effects by activating cell cycle arrest, inducing apoptosis and autophagy, inhibiting angiogenesis, and increasing the generation of reactive oxygen species to induce oxidative stress, which all contribute to cancer cell death [5,6].

Nutrition has been shown to mediate epigenetic mechanisms, and recent studies have demonstrated that both the quantity and quality of food intake are associated with aging and cancer incidence and prognosis. Moreover, nutrition is thought to be the most influential of all external environmental factors due to its ability to affect the transcriptional activity and expression of specific genes [1]. Natural products have received increasing attention in recent years as novel anticancer agents, and interest in the potential chemopreventive and therapeutic properties of food-derived compounds, including plant polyphenols, has increased tremendously. Several members of phytochemicals are able to inhibit tumour growth as well as carcinogenesis at many points, and are also potential natural therapeutic agents. They may inhibit the activation of several signalling pathways, such as *PI3K/AKT*, *MAPK/ERK* and *JAK/STAT3*, thereby ultimately inhibiting tumour growth and metastasis, and may also stimulate ROS-mediated apoptosis and autophagy [7,8,9,10]. Polyphenols have a full range of anti-tumour effects, including, in addition to the above, inhibition of angiogenesis and activation of the immune system [11].

Plant polyphenols can be found in many fruits and vegetables, including soy, turmeric, grapes, celery, apples, onions, parsley, bell peppers, green tea, and black pepper, and have been shown to possess chemopreventive activity [1]. Natural compounds found in plants have demonstrated potential HDACi activity, including trans-resveratrol found characteristically in grapes, red wine, blueberries, and peanuts as main active ingredients [6]. HDACis of both natural and synthetic origins are currently being evaluated in clinical trials to determine their antitumor efficacies and potential side effects. 7,12-Dimethylbenz[a]anthracene (DMBA) is a known carcinogen that is used as a general compound in in vitro studies focusing not only on tumour models, but also on epigenetic studies [12,13,14,15]. To study the effect of the carcinogenic compound DMBA on early gene expression modification, a 24 h treatment seems to be optimal in an animal model, as many genes involved in the cell regulation respond by this time point [16,17].

Our study aimed to explore whether the frequent consumption of aqueous extract of a fruit seed and peel extract rich in phytochemicals influences the expression levels of *DNMT* and *HDAC* genes, which are responsible for the epigenetic changes that affect tumour formation and prevention. Specifically, we tested whether an extract derived from fruit seeds, lyophilized shells, and dried red grapes, blackberries, blackcurrants, redcurrants, and rosehips under the brand name Fruit Café (ProVitamix, Budapest, Hungary), which contains 100–200 mg/portion/day of polyphenols/flavonoids, could prevent the early stages of tumour formation following carcinogenic DMBA exposure by modulating *DNMT* and *HDAC* mRNA levels. Detection of trans-resveratrol and its glucoside trans-piceid was also performed. Our study showed that the tested polyphenol-rich extract, which provides dietary levels of polyphenols, can effectively reduce the expression of *DNMT* and *HDAC* genes.

## 2. Materials and Methods

### 2.1. HPLC Measurements

*Chemicals and reagents:* The *trans*-resveratrol standard (99%) was purchased from Sigma-Aldrich Co. (Budapest, Hungary), the *trans*-piceid standard was purchased from Herbstandard Inc. (Chesterfield, MO, USA), acetic acid (96%) was obtained from Riedel-de Haën GmbH & Co. (Seelze, Germany) and methanol (HPLC grade) was acquired from Scharlau Chemie S.A. (Barcelona, Spain).

**Figure 1:**
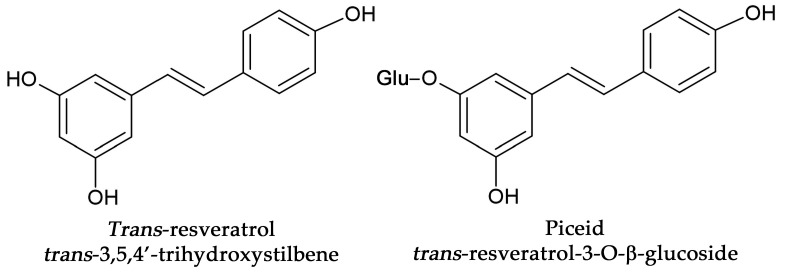
Structure of *trans*-resveratrol and *trans*-piceid.

*Standard solutions and sample preparation:* The standards were dissolved in a small portion of ethanol and filled up with the eluent. The fruit extract samples were prepared in a coffee machine with tap water as it is described in “Treatments of animals” section to obtain 100 mL of solution per portion, filtrated by using Millex syringe filter (Millipore Kft., Budapest, Hungary) and injected. All standard solutions and fruit extract samples were stored in the dark place at 5 °C to avoid oxidative degradation and isomerisation of *trans*-resveratrol and *trans*-piceid to *cis*-configuration (Figure 1).

*HPLC instrumentation and conditions:* The system used for high-performance liquid chromatography (HPLC) consisted of a Gynkotek M 580 GT pump, a Rheodyne 8125 (20 μL loop) injector and a Gynkotek M 340S UV diode array detector (Gynkotek GmbH, Germering, Germany). A 250 × 4.6 mm column packed with 6 μm particle size C18 material has been used for the separations. A Chromeleon data management software (Dionex Corp., Sunnyvale, CA, USA) was used for the control of the equipment and for data evaluation. Quantization was carried out using peak areas method. A multistep gradient method was applied using methanol–water–acetic acid (10:90:1 *v*/*v*) mixture as solvent A and methanol–water–acetic acid (90:10:1 *v*/*v*) mixture as solvent B at a flow rate of 1.5 cm^3^ min^−1^. The gradient profile was 0.0–18.0 min from 0% to 40% B; 18.0–25.0 min from 40% to 100% B; 25.0–27.0 min 100% B. Chromatographic separations were monitored at 306 nm. Chromatographic peaks were identified by comparing retentions and UV spectra and MS spectra of the samples with those of the standard compounds. Quantisation was carried out by external standardisation.

*MS instrumentation and conditions:* MS (mass spectrometry) analysis was performed using a Finnigan AQA (Thermoquest, San Jose, CA, USA) mass spectrometer. The auxiliary and the curtain gas were nitrogen at the flow rate of 600 L/h. For HPLC-MS analysis, we used ESI (electrospray ionization) source, the probe temperature was 250 °C, the probe voltage was 3.5 kV. Spectra were recorded by 1.2 scan/s in the negative ion mode between *m*/*z* 10 and 700. The scan filter on the quadrupole analyser was 10 and 20 V. Finnigan Xcalibur (version: XCALI-97006) was used to acquire the mass spectra of the compounds.

### 2.2. Animals

Male CD1 mice (Charles River Laboratories International, Budapest, Hungary) were housed separately in polycarbonate cages and maintained in a 12:12 h light–dark cycle, provided with water and standard pellets ad libitum throughout the experiment. Four groups of 6-week-old (18 ± 2 g) male CD1 mice were used for this study, with six animals in each group.

### 2.3. Treatment of Animals

The negative control group (Control group) was provided with standard feed and tap water ad libitum. The positive control group (DMBA group) was provided with standard feed and tap water ad libitum, and 20 mg/kg body weight (bw) DMBA (Sigma-Aldrich, Budapest, Hungary), dissolved 0.2 mL corn oil, was administered intraperitoneally 24 h before cervical dislocation. The third group (FC group) received drinking water containing a human-equivalent dose of the fruit seed and peel extract for 28 days, in addition to ad libitum access to standard feed. The fourth group (FC + DMBA group) received drinking water containing a human-equivalent dose of the fruit seed and peel extract for 28 days, in addition to ad libitum access to standard feed, and 20 mg/kg bw DMBA, dissolved in 0.2 mL corn oil, was administered intraperitoneally 24 h before cervical dislocation (Table 1). The animals in first and third groups received 0.2 mL corn oil vehicle intraperitoneally. The fruit seed and peel extract contained lyophilized seeds, the skins of red grape, blackcurrant, redcurrant, rosehip, and black cherry, and fructose [13,14,18] commercialized under the brand name Fruit Café (ProVitamix, Budapest, Hungary). The extract was prepared in a regular high-pressure coffee from two components. One component is the seed extract and the other one is peel extract. A total of 60 doses from 1000 g of seed extract and 700 g of peel extract can be produced, and 8 mL of the 100 mL solution was dissolved in tap water to a final volume of 40 mL, which generated a daily dose for 12 mice, equivalent to the human dose [19].

Animals were autopsied after euthanasia was performed by cervical dislocation. All animals received humane care, and the experimental protocol received ethical approval.

### 2.4. Total RNA Isolation

During the autopsy, the liver and kidneys were collected from every animal and total mRNA was isolated using TRIzol reagent (MRTR118-20, NucleotestBio, Budapest, Hungary) according to the manufacturer’s protocol.

Tissue samples were homogenised with TRIzol reagent (1 mL per 100 mg tissue) using a Janke and Kunkel Ultra Turrax T25 stirrer (IKA-Werke, Staufen, Germany), and 100 µL of chloroform (Merck, Budapest, Hungary) was added to the TRIzol lysate and mixed thoroughly by shaking. After 5 min at room temperature, the samples were centrifuged at 12,000× *g* for 15 min at 4 °C, and the upper aqueous phase containing RNA was collected in a new Eppendorf tube.

Then, 250 µL of isopropanol (Merck, Budapest, Hungary) was added to the sample, mixed and kept at room temperature for 10 min before centrifugation at 12,000× *g* at 4 °C for 10 min. The supernatant was discarded and the pellet was washed with 70% ethanol (Merck, Budapest, Hungary) and then centrifuged at 7500× *g* at 4 °C for 5 min. The pellet was air-dried and RNase-free water (Merck, Budapest, Hungary) was added. Finally, the RNA was quantified with a MaestroNano (Maestrogen, Hsinchu City, Taiwan) spectrophotometer.

### 2.5. qRT-PCR

The assays were performed on the Roche 480 instrument (Roche, Budapest, Hungary) using the KAPA SYBR FAST One-Step qRT-PCR Master Mix Kit (Sigma-Aldrich, Budapest, Hungary). The amplifications were performed in 20 μL reaction volume, mixing 5 μL RNA target (100 ng) and 15 μL of master mix with forward and reverse primers (10 µL KAPA SYBR FASTqPCR Master Mix, 0.4 µL KAPA RT Mix, 0.4 µL dUTP, 0.4 µL primer (200 nM), 3.8 µL sterile double distilled water). The reactions were performed using the following thermal profile: 42 °C for 5 min for the reverse transcription step, a hot-start denaturation step of 95 °C for 3 min, followed by 45 cycles of 95 °C for 10 s (denaturation), 60 °C for 20 s (annealing/extension). The fluorogenic signal emitted during the annealing/extension step was read and analysed using the software. Immediately after amplification, a melting curve protocol was generated by increasing each cycle by 0.5 °C for 80 cycles of 10 s each, starting from the target temperature (55.0 °C).

The primary sequences of the housekeeping gene used as an internal control, hypoxanthine phosphoribosyltransferase 1 (*HPRT1*), and the genes of interest, *DNMT1*, *DNMT3A*, *DNMT3B*, *HDAC2*, *HDAC3* and *HDAC8*, are listed in Table 2. Primers were designed using Primer Express™ software (Applied Biosystems, Budapest, Hungary) and synthesised by Integrated DNA Technologies (Bio-Sciences, Budapest, Hungary). The results were analysed using the relative quantification method (2^-∆∆CT^).

### 2.6. Statistical Analysis

The Kolmogorov–Smirnov test was used to verify the normality of the results. Means between groups were compared using analysis of variance (ANOVA). Statistical analyses were performed using IBM SPSS Statistics for Windows, Version 26.0 (Armonk, NY, USA). Significance was established at *p* < 0.05.

## 3. Results

**Figure 2:**
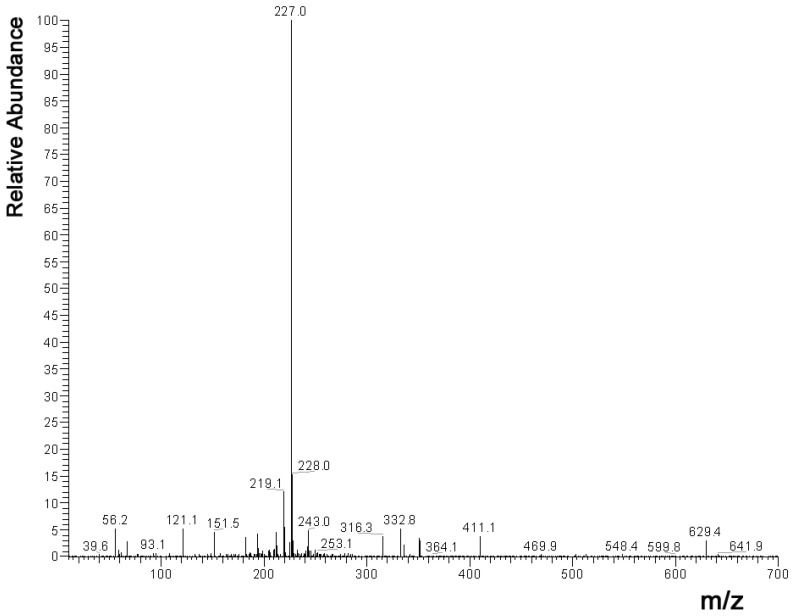
ESI negative ion MS spectra of trans-resveratrol. The negative polarised quasi-molecular ion derived from trans-resveratrol was detected at 227.0 *m*/*z* (ESI = electrospray ionization, MS = mass spectrometry).

**Figure 3:**
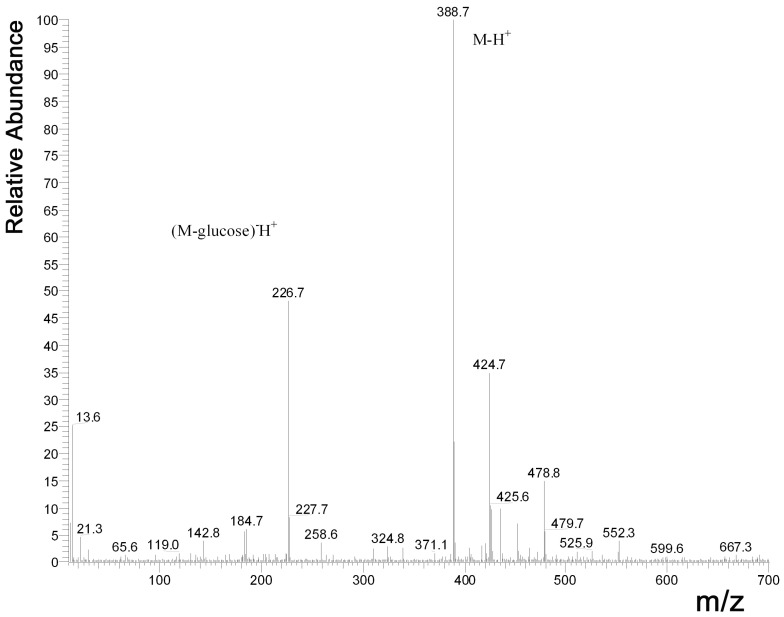
ESI negative ion MS spectra of quasi-molecular trans-piceid at 388.7 *m*/*z*. The peak of trans-resveratrol, which was partially presented in native form and generated through the fragmentation of piceid precursor as well, was detected at 226.7 *m*/*z* (ESI = electrospray ionization, MS = mass spectrometry).

After analysis of the fruit extract by HPLC, the calculated amount of trans-resveratrol (Figure 2) was 1.74 mg/L (SD 0.13) and that of trans-piceid (Figure 3) was 2.37 mg/L (SD 0.32). Therefore, the mean daily intake of trans-resveratrol was 5.8 μg, while the mean daily intake of trans-piceid was 7.9 μg for each mouse, respectively. Using the metabolic multiplier between humans and mice (12.3×), this would result in a human consuming 366.76 micrograms of trans-resveratrol and 499.55 micrograms of trans-piceid daily.

We tested the efficacy of a fruit seed and peel extract to attenuate *HDAC* and *DNMT* gene expression following the administration of the carcinogen DMBA in an animal model.

**Figure 4:**
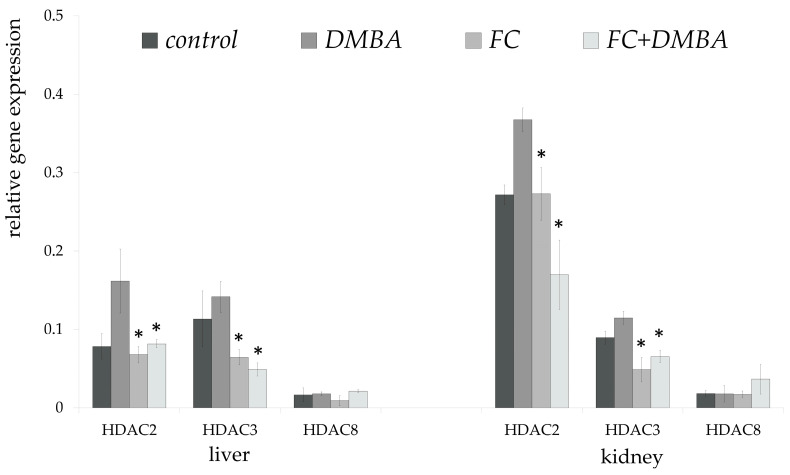
Relative gene expression of *HDAC* genes. The relative gene expression levels of histone deacetylases at the mRNA level in the liver and kidney of experimental animals relative to the mRNA levels of *HPRT1* (DMBA: 7,12-dimethylbenz(a)anthracene, HPcFE: fruit seed and peel extract); * significantly different from DMBA-treated mice (*p* < 0.05). Error bars represent the standard deviation.

We measured the relative gene expression of *HDAC2*, *HDAC3*, and *HDAC8* in both liver and kidney isolates. DMBA administration significantly increased the relative expression of *HDAC2* in the liver of the DMBA group twofold compared to the Control group; however, the fruit seed and peel extract completely diminished the effects of DMBA (Figure 4). The relative gene expression pattern of *HDAC3* in the liver was similar to that observed for *HDAC2*; however, gene expression of both genes was reduced threefold in the FC group compared with that in the Control group. No significant changes were observed for *HDAC8* expression. A similar trend was observed in the kidney for all three genes (Figure 4).

In the liver, fruit and peel extract significantly reduced the expression of the genes tested not only in those elevated by DMBA, but also in the control groups. However, the most significant reduction occurred in the FC + DMBA groups. FC induced a more than tenfold, 3.5-fold and 3-fold decrease in the expression of *DNMT1*, *DNMT3A* and *DNMT3B* genes, respectively, compared to the DMBA groups.

**Figure 5:**
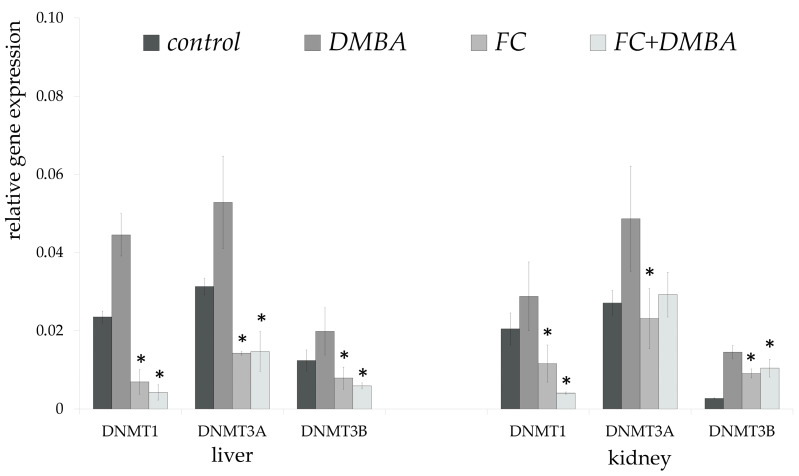
Relative gene expression of *DNMT* genes. Relative gene expression of DNA methyltransferases at the mRNA level in the liver and kidney of experimental animals compared with the mRNA level of *HPRT1* (DMBA: 7,12-dimethylbenz(a)anthracene, HPcFE: Fruit seed and peel extract); * significantly different from DMBA-treated mice (*p* < 0.05). Error bars represent the standard deviation.

In the kidneys, DMBA exposure significantly increased the gene expression of *DNMT3A* and *DNMT3B* compared with the control. The fruit seed and peel extract reduced this change in gene expression but less emphatically than in the liver (Figure 5).

## 4. Discussion

In our animal model, DMBA was used as a carcinogenic compound, and we analysed whether pre-treatment with a commercially available fruit seed and peel extract exerted chemopreventive and cellular regulatory function in an animal model. Our results revealed, in general, that the fruit and peel extract, which is rich in polyphenols and flavonoids, protected against DMBA-induced alterations in gene expression in the liver and kidneys. DMBA treatment significantly increased the expression of most examined *DNMT* and *HDAC* genes, whereas pre-treatment with the fruit seed and peel extract decreased the expression of these genes, even relative to the Control group.

Based on our HPLC measurements, the recommended daily intake of the fruit and peel extract contains trans-resveratrol and trans-piceid in amounts equivalent to the mean consumption in the adult Spanish population. This amount corresponds to 0.2 L of red wine for trans-resveratrol and 1 L of red wine for trans-piceid. This population in the lowest quartile consumes only a fraction of the mean [20]. Considering that resveratrol is mainly found in red wine, consumption of similar products tested could significantly increase resveratrol intake in the population without red wine consumption. The HPLC measurement also indicated that the animals received amounts of resveratrol close to the mean human intake. Similar animal studies carried out by us are very rare. The studies focus mainly on tolerance, toxicity, renal and hepatic damage and the effects of high doses in general [21].

Histone acetylation, which is regulated by *HDAC* activity [4], has been shown to modulate gene expression, and the post-transcriptional modifications of acetylation and methylation may play roles in cancer development by regulating the expression of tumour suppressor genes and oncogenes. Our results demonstrate that the fruit seed and peel extract significantly decreased *HDAC2* and *HDAC3* expression in both the liver and kidney compared with the Control group, which consumed only standard laboratory pellets, and with the DMBA-treated group. However, no significant changes in *HDAC8* expression were observed in either the liver or kidney.

There may be a special case where DMBA combined with fruit and peel extract results in lower expression than fruit and peel extract alone. Such examples are shown in Figure 4 for *HDAC3* in the liver and *HDAC2* in the kidney. Similar observations have been made by other researchers, but this may be a gene- and tissue-dependent issue, and not a universal one. For example, Singhal and colleagues investigated the effect of oestrogen modification on DMBA carcinogenesis in the liver of ovariectomised rats. They determined that the expression of *CYP1A1* and *CYP1B1* was higher in the co-administration of oestrogen and DMBA than in the DMBA group. The expression of *NQO1* was increased by DMBA, but this increase was more modest in the presence of DMBA and oestrogen together. For *GSTM3*, DMBA and oestrogen resulted in lower expression than DMBA or oestrogen alone [20].

Mirza et al. described results, similar to our findings, for the relative gene expression levels of *DNMT1*, *DNMT3A*, and *DNMT3B* measured by RT-PCR in breast cancer cells treated with multiple natural chemopreventive substances, such as EGCG, genistein, withaferin A, curcumin, resveratrol, and guggulsterone. These natural substances significantly decreased *DNMT* gene expression, suggesting potential chemopreventive activities. *DNMT*s are recognized as major enzymes involved in the somatic inheritance of DNA methylation, playing crucial roles in epigenome maintenance. The upregulation of the DNMT1 expression may induce the dissociation of p21WAF1 from PCNA, which may make p21WAF1 more vulnerable to ubiquitination and proteasomal degradation [22]. Supplementation with the fruit seed and peel extract in our experiment resulted in relatively low gene expression levels of *DNMT1* and *DMNT3A* in both the kidneys and liver compared with the Control group and prevented increased gene expression following DMBA exposure, whereas *DNMT3B* only showed lower levels in the FC groups compared to the DMBA groups.

The chemopreventive effects of fruit seed and peel extracts, which contain polyphenols and flavonoids, have been demonstrated in several studies. Flavonoids, such as anthocyanin and luteolin, are abundant in many fruits and vegetables. Ursolic acid, a natural triterpenoid found in blueberries and cranberries, activates the nuclear factor-erythroid factor 2–related factor 2 (*Nrf2*) pathway and decreases the enzymatic activities of DNMTs and HDACs, leading to chemopreventive and antitumor activities [23,24,25].

Various studies have demonstrated that DNA methylation, which is regulated by the histone acetylation status, results in gene silencing in breast cancer cells. Several hypermethylated genes have been associated with gene silencing, such as the cell cycle inhibitor genes *CDKN2A* (*p16*) and *RASSF1A*, and the DNA repair gene *BRCA1*. The combination of sulforaphane (SFN) with the main polyphenol found in green tea, epigallocatechin-3-gallate (EGCG), can reactivate oestrogen receptor (ER)-α expression in ER-α-negative breast cancer cell lines, restoring sensitivity to antioestrogen treatment [26,27]. Therefore, the inhibition of HDAC and DNMT activities may provide new treatment options for patients with ER-α-negative breast cancer [23]. According to a recent study, the carcinogenic polycyclic aromatic hydrocarbons, such as benzofluorene and benzo (a) pyrene (BaP), can significantly increase the enzymatic levels of DNMT1l [28].

Recently, black raspberry-derived anthocyanins, a subclass of flavonoids, have been demonstrated to suppress the enzymatic activities and protein levels of DNMT1 and DNMT3B in multiple colon cancer cell lines, including HCT116, Caco-2, and SW480 cells leading to demethylation. The demethylation of promoters for cyclin-dependent kinase inhibitor 2A (*CDKN2A*) and secreted frizzled-related protein 2 (*SFRP2*), in addition to *SFRP5* and Wnt inhibitory factor 1 (*WIF1*), which are upstream of the *Wnt* signaling pathway, results in the increased mRNA expression of these genes. The mRNA expression genes downstream of the *Wnt* pathway, including *β-catenin* and *c-Myc*, also decreased, resulting in reduced cell proliferation and increased apoptosis [29]. Resveratrol, an active ingredient found in red grapes, peanuts, and berries, has been linked to health and disease prevention due to reported cardioprotective, antioxidative, anti-inflammatory, and anticancer activities [26]. Resveratrol decreases *DNMT1* and *DNMT3B* expression levels and modulates the aberrant expression profiles of microRNAs (miRNAs) in cancer cell lines and tumour tissues [30].

Trans-resveratrol can reduce the occurrence of Ras association domain family 1 isoform A (*RASSF1A*) hypermethylation by inhibiting *DNMT* activity in women with increased breast cancer risk [31,32]. Pterostilbene is a dimethyl ether derivative of resveratrol found in blueberries, grapes, and herbs, such as *Pterocarpus indicus*. By suppressing the activities of Sirtuin 1 (*SIRT1*) and *DNMT*s, resveratrol and pterostilbene can synergistically inhibit cell viability, induce apoptosis, and arrest the cell cycle in triple-negative breast cancer cells, inhibiting telomerase activity and histone *H2AX* expression [3,5]. In summary, DNMT and HDAC inhibitors target several pathways that contribute to cancer development [33].

Overall, it can be assumed that the increase in carcinogenic DMBA-induced expression of *HDAC1*, *HDAC2*, *DNMT1*, *DNMT3A* and *DNMT3B* genes tested was reduced in most cases by the fruit peel and seed extract tested. This finding is significant because normal dietary conditions were modelled in our studies. However, it provides the amount that already has a significant protective effect against many diseases [34,35].

## 5. Conclusions

The results of this study indicated that fruit and peel extract administered to mice at human-equivalent doses protected against the DMBA-induced upregulation of *DNMT* and *HDAC* genes, which was likely due to the chemopreventive effects of the various compounds found in fruit seed and peel extract, which is rich in polyphenols and flavonoids. Consumption of one serving of the fruit seeds and peel extracts tested does not exceed the regular daily intake of polyphenols, trans-resveratrol and trans-piceid. Although vast amounts of data are available in the literature associated with specific gene expression, relatively few research groups have examined the epigenetic effects of natural extracts in normal dietary intake level in animal models. Our results suggest that an extract derived from fruit peels and seeds provides effective protection against early gene expression changes of genes involved in epigenetic processes induced by the carcinogen DMBA.

## Figures and Tables

**Table 1:** Treatment program.

		Fluid Consumption (for 28 Days)		Carcinogenic Exposure (on Day 27) **	
Group 1	Control	Tap water	ad libitum	Vehicle	
Group 2	DMBA	Tap water	ad libitum	DMBA	20 mg/kg bw
Group 3	FC	Fruit seed and peel extract	Human-equivalent dose *	Vehicle	
Group 4	FC + DMBA	Fruit seed and peel extract	Human-equivalent dose *	DMBA	20 mg/kg bw

The experimental animals received ad libitum water after full consumption of the polyphenolic fruit seed and peel extract (FC; *) and purified corn oil as the vehicle or DMBA (7,12-dimethylbenz(a)anthracene) dissolved in purified corn oil was administered intraperitoneally 24 h prior to cervical dislocation and dissection (**).

**Table 2:** PCR primers.

Gene Name	Forward Primer	Reverse Primer
DNA methyltransferase 1 (*DNMT1*)	F-AAGAATGGTGTTGTCTACCGAC	R-CATCCAGGTTGCTCCCCTTG
DNA methyltransferase 3A (*DNMT3A*)	F-GAGGGAACTGAGACCCCAC	R-CTGGAAGGTGAGTCTTGGCA
DNA methyltransferase 3B (*DNMT3B*)	F-AGCGGGTATGAGGAGTGCAT	R-GGGAGCATCCTTCGTGTCTG
Histone deacetylase 2 (*HDAC2*)	F-GGAGGAGGCTACACAATCCG	R-TCTGGAGTGTTCTGGTTTGTCA
Histone deacetylase 3 (*HDAC3*)	F-GCCAAGACCGTGGCGTATT	R-GTCCAGCTCCATAGTGGAAGT
Histone deacetylase 8 (*HDAC8*)	F-ACTATTGCCGGAGATCCAATGT	R-CCTCCTAAAATCAGAGTTGCCAG
Hypoxanthine phosphoribo-syltransferase 1 (*HPRT1*)	F-TCAGTCAACGGGGGACATAAA	R-GGGGCTGTACTGCTTAACCAG

Sequences of primers used for qRT-PCR are listed.

## Data Availability

The data presented in this study are available in this article and in the Supplementary Materials.

## Supplementary Materials

The following supporting information can be downloaded at: https://www.mdpi.com/article/10.3390/nu15081867/s1, Dataset: Original measurement data and calculations.
